# Supplementary material for: Characterization of a Mn-SOD from the desert beetle Microdera punctipennis and its increased resistance to cold stress in E. coli cells
Source: PeerJ. 2020 Feb 14;8:e8507. doi: 10.7717/peerj.8507 (PMC7025704; doi:10.7717/peerj.8507)
Supplement: Supplemental Information 1 — The beetle individuals were exposed at 4 °C for different time periods (0.5 h, 1 h, 1.5 h, 2 h, 3 h, 5 h, 7 h, 9 h and 11 h, respectively, three replicates per treatment group). The individuals at room temperature (about 25 °C) without any cold treatment were used as control. Translation elongation factor (EF- α) was used as a reference gene to normalize the target gene expression levels among samples. The relative expression of the target gene was calculated using the comparative 2−△△CT method. The change of the gene expression levels at 4° was normalized to the gene in the control at 25°. [file peerj-08-8507-s001.docx]

| 0h | 0.5h | 1h | 1.5h | 2h | 3h | 5h | 7h | 9h | 11h |
| --- | --- | --- | --- | --- | --- | --- | --- | --- | --- |
| 1 | 9.352 | 29.9735 | 142.9959 | 0.8032 | 18.1153 | 10.3768 | 7.3745 | 0.5974 | 74.8056 |
| 1 | 11.1437 | 27.2651 | 112.241 | 0.1454 | 18.2435 | 11.2981 | 6.9335 | 0.5426 | 60.9517 |
| 1 | 9.5778 | 16.7642 | 108.2167 | 1.4495 | 12.355 | 9.7214 | 10.0661 | 0.1111 | 67.1982 |

**Supplementary data. S1. The relative expression data of *MpmMn-SOD*.** The beetle individuals were exposed at 4 ℃ for different time periods (0.5 h, 1 h, 1.5 h, 2 h, 3 h, 5 h, 7 h, 9 h and 11 h, respectively, three replicates per treatment group). The individuals at room temperature (about 25 ℃) without any cold treatment were used as control. Translation elongation factor (*EF-α*) was used as a reference gene to normalize the target gene expression levels among samples. The relative expression of the target gene was calculated using the comparative 2^-△△CT^ method. The change of the gene expression levels at 4 ℃ was normalized to the gene in the control at 25 ℃.
