# Supplementary material for: Characterization of a Mn-SOD from the desert beetle Microdera punctipennis and its increased resistance to cold stress in E. coli cells
Source: PeerJ. 2020 Feb 14;8:e8507. doi: 10.7717/peerj.8507 (PMC7025704; doi:10.7717/peerj.8507)
Supplement: Supplemental Information 2 — Adult beetles were dissected in cold 1 ×PBS to isolate different tissues, such as head, midgut, hindgut (containing Malpighian tubule), fat body and carcass (whole body after head, gut and fat body were removed). Translation elongation factor (EF- α) was used as a reference gene to normalize the target gene expression levels among samples. The relative expression of the target gene was calculated using the comparative 2−△△CT method. [file peerj-08-8507-s002.docx]

| Head | Midgut | Hindgut | Fat body | Carcass |
| --- | --- | --- | --- | --- |
| 0.978287 | 5.565409 | 67.41418 | 19.96725 | 3.620755 |
| 0.511387 | 6.370702 | 51.77858 | 15.50592 | 3.495587 |
| 2 | 5.840871 | 54.70448 | 17.1121 | 3.64649 |

**Supplementary data. S2.** **Tissue distribution datas of *MpmMn-SOD.*** Adult beetles were dissected in cold 1×PBS to isolate different tissues, such as head, midgut, hindgut (containing Malpighian tubule), fat body and carcass (whole body after head, gut and fat body were removed). Translation elongation factor (*EF-α*) was used as a reference gene to normalize the target gene expression levels among samples. The relative expression of the target gene was calculated using the comparative 2^-△△CT^ method.
