# Supplementary material for: Characterization of a Mn-SOD from the desert beetle Microdera punctipennis and its increased resistance to cold stress in E. coli cells
Source: PeerJ. 2020 Feb 14;8:e8507. doi: 10.7717/peerj.8507 (PMC7025704; doi:10.7717/peerj.8507)
Supplement: Supplemental Information 3 — Bacteria cultures were induced to produce target protein Trx-His-MpmMn-SOD by addition of IPTG followed by additional 10 h incubation at 25 °C and 5 mL cultures of the bacteria were exposed to −4 °C for 0 h, 2 h, 4 h and 6 h, respectively. The control group was at −4 °C for 0 h, in which the OD595 of the cultures were determined without cold treatment. Each treatment had three replicates. The supernatants were collected as crude enzyme liquids and were quantified using the BCA Protein Assay Kit (Thermo Scientific Pierce, IL, USA). The SOD activity was detected using the CuZn/Mn-SOD Assay Kit (Jiancheng, Nanjing, China) following the manufacturer’s protocol. The increase of SOD activity was calculated by subtracting the SOD activity of BL21(pET-32a-MpmMn-SOD) from that of the control BL21(pET-32a), then dividing the difference by the SOD activity of BL21(pET-32a-MpmMn-SOD). [file peerj-08-8507-s003.docx]

**A**

|  | BL21(pET32a) | | | BL21(pET32a-Mn-SOD) | | |
| --- | --- | --- | --- | --- | --- | --- |
| 0h | 2.454404 | 2.197547 | 2.383055 | 3.782703 | 3.71595 | 3.693698 |
| 2h | 2.367381 | 2.122817 | 2.142382 | 4.976881 | 5.057587 | 5.272804 |
| 4h | 2.933457 | 2.893993 | 2.867684 | 5.023548 | 5.090529 | 5.090529 |
| 6h | 1.887328 | 1.914035 | 1.914035 | 3.938462 | 3.963232 | 4.111853 |

**B**

| Relative increase in SOD activity | |
| --- | --- |
| 0h | 0.594043 |
| 2h | 1.315316 |
| 4h | 0.748878 |
| 6h | 1.101891 |

**Supplementary data. S3.** **A.** **SOD activity (U/mg protein) in bacteria BL21 (pET32a) and (pET32a-mMn-SOD) and B. Relative increase in SOD activity.** Bacteria cultures were induced to produce target protein Trx-His-MpmMn-SOD by addition of IPTG followed by additional 10 h incubation at 25 ℃, and 5 mL cultures of the bacteria were exposed to -4 ℃ for 0 h, 2 h, 4 h and 6 h, respectively. The control group was at -4 ℃ for 0 h, in which the OD595 of the cultures were determined without cold treatment. Each treatment had three replicates. The supernatants were collected as crude enzyme liquids and were quantified using the BCA Protein Assay Kit (Thermo Scientific Pierce, IL, USA). The SOD activity was detected using the CuZn/Mn-SOD Assay Kit (Jiancheng, Nanjing, China) following the manufacturer’s protocol. The increase of SOD activity was calculated by subtracting the SOD activity of BL21(pET-32a-MpmMn-SOD) from that of the control BL21(pET-32a), then dividing the difference by the SOD activity of BL21(pET-32a-MpmMn-SOD).
