# Supplementary material for: Characterization of a Mn-SOD from the desert beetle Microdera punctipennis and its increased resistance to cold stress in E. coli cells
Source: PeerJ. 2020 Feb 14;8:e8507. doi: 10.7717/peerj.8507 (PMC7025704; doi:10.7717/peerj.8507)
Supplement: Supplemental Information 4 — Bacteria cultures were induced to produce target protein Trx-His-MpmMn-SOD by addition of IPTG followed by additional 10 h incubation at 25 °C and 5 mL cultures of the bacteria were exposed to −4 °C for 0 h, 2 h, 4 h and 6 h, respectively. The control group was at −4 °C for 0 h, in which the OD595 of the cultures were determined without cold treatment. Each treatment had three replicates. The supernatants were collected as crude enzyme liquids and were quantified using the BCA Protein Assay Kit (Thermo Scientific Pierce, IL, USA). O2•− content was measured according to the hydroxylamine oxidation method. The decrease in O2•− content was calculated by subtracting the O2•− content of BL21(pET-32a-MpmMn-SOD) from that of the control BL21(pET-32a), then dividing the difference by the O2•− content of BL21(pET-32a-MpmMn-SOD). [file peerj-08-8507-s004.docx]

**A.**

|  | BL21(pET32a) | | | BL21(pET32a-Mn-SOD) | | |
| --- | --- | --- | --- | --- | --- | --- |
| 0h | 71.5127 | 70.416 | 67.1259 | 52.8688 | 50.6754 | 50.6754 |
| 2h | 60.5457 | 58.3523 | 60.5457 | 45.1919 | 44.0952 | 44.0952 |
| 4h | 63.8358 | 64.9325 | 67.1259 | 50.6754 | 53.9655 | 57.2556 |
| 6h | 63.8358 | 61.6424 | 62.7391 | 51.7721 | 48.482 | 48.482 |

**B.**

| Relative decrease in O_2_·^-^ content | |
| --- | --- |
| 0h | 0.26204 |
| 2h | 0.256541 |
| 4h | 0.174033 |
| 6h | 0.209907 |

**Supplementary data. S4. A. O_2_•^-^ content (umol/ml)** **in bacteria BL21 (pET32a) and (pET32a-mMn-SOD) and B. Relative decrease in O_2_•^-^ content.** Bacteria cultures were induced to produce target protein Trx-His-MpmMn-SOD by addition of IPTG followed by additional 10 h incubation at 25 ℃, and 5 mL cultures of the bacteria were exposed to -4 ℃ for 0 h, 2 h, 4 h and 6 h, respectively. The control group was at -4 ℃ for 0 h, in which the OD595 of the cultures were determined without cold treatment. Each treatment had three replicates. The supernatants were collected as crude enzyme liquids and were quantified using the BCA Protein Assay Kit (Thermo Scientific Pierce, IL, USA). O_2_•^-^ content was measured according to the hydroxylamine oxidation method. The decrease in O_2_•^-^ content was calculated by subtracting the O_2_•^-^ content of BL21(pET-32a-MpmMn-SOD) from that of the control BL21(pET-32a), then dividing the difference by the O_2_•^-^ content of BL21(pET-32a-MpmMn-SOD).
