# Supplementary material for: Characterization of a Mn-SOD from the desert beetle Microdera punctipennis and its increased resistance to cold stress in E. coli cells
Source: PeerJ. 2020 Feb 14;8:e8507. doi: 10.7717/peerj.8507 (PMC7025704; doi:10.7717/peerj.8507)
Supplement: Supplemental Information 5 — Inhibited zones of the bacteria on agar plates. Different H2O2 concentrations from 25 mM to 100 mM. mMn-SOD: E. coli cells BL21(pET32a-mMn-SOD); Control: E. coli cells BL21(pET32a). Observe the zone of inhibition formed around the cup , and record the diameter of the zone. Values are compared to the control bacteria between the same group. The data are the mean ± S.E. of three replicates. [file peerj-08-8507-s005.docx]

| Hydroperoxide Contentration(mM) | BL21(pET32a) | | | BL21(pET32a-Mn-SOD) | | |
| --- | --- | --- | --- | --- | --- | --- |
| 25 | 2.332 | 2.661 | 2.672 | 1.855 | 2.202 | 2.206 |
| 50 | 2.932 | 2.928 | 3.086 | 2.149 | 2.632 | 2.64 |
| 75 | 3.087 | 3.123 | 3.249 | 2.398 | 2.800 | 2.769 |
| 100 | 3.402 | 3.413 | 3.468 | 2.71 | 3.006 | 3.026 |

**Supplementary data. S5. Diameter of inhibition zone around the Oxford Cup (cm).** Inhibited zones of the bacteria on agar plates. Different H_2_O_2_ concentrations from 25 mM to 100 mM. mMn-SOD: *E. coli* cells BL21(pET32a-mMn-SOD); Control: *E. coli* cells BL21(pET32a). Observe the zone of inhibition formed around the cup, and record the diameter of the zone. Values are compared to the control bacteria between the same group. The data are the mean ± *S.E.* of three replicates.
