# Supplementary material for: Characterization of a Mn-SOD from the desert beetle Microdera punctipennis and its increased resistance to cold stress in E. coli cells
Source: PeerJ. 2020 Feb 14;8:e8507. doi: 10.7717/peerj.8507 (PMC7025704; doi:10.7717/peerj.8507)
Supplement: Supplemental Information 6 — The bacterial cells were washed with 5% dextrose solution until the bacterial solution’s relative conductivity was comparable to that of 5% glucose solution (denoted as L1). The isotonic bacterial solutions of 5 mL were exposed to −4 °C for 0 h, 2 h, 4 h and 6 h, respectively, and the relative conductivity was measured (denoted as L2). After boiling for 5 min, the relative conductivity of the treated bacteria solution was measured again (denoted as L0). The relative conductivity of the bacterial membrane permeability is calculated as: Relative conductivity (%) = 100 × (L2 -L1)/L0. Paired t-test was used to analyze the difference between BL21 (pET32a-mMn-SOD) and BL21 (pET32a) in each concentration group. The symbols * indicate statistical significance, P < 0.05. Values are expressed as means ± S.E. (n = 3). [file peerj-08-8507-s006.docx]

|  | BL21(pET32a) | | | BL21(pET32a-Mn-SOD) | | |
| --- | --- | --- | --- | --- | --- | --- |
| 0h | 0.36 | 0.44 | 0.36 | 0.4 | 0.32 | 0.44 |
| 2h | 0.46 | 0.52 | 0.68 | 0.4 | 0.36 | 0.48 |
| 4h | 0.52 | 0.64 | 0.68 | 0.42 | 0.4 | 0.48 |
| 6h | 0.68 | 0.64 | 0.8 | 0.48 | 0.48 | 0.54 |

**Supplementary data. S6. Relative electrical conductivity data.** The bacterial cells were washed with 5% dextrose solution until the bacterial solution's relative conductivity was comparable to that of 5% glucose solution (denoted as L1). The isotonic bacterial solutions of 5 mL were exposed to −4 °C for 0 h, 2 h, 4 h and 6 h, respectively, and the relative conductivity was measured (denoted as L2). After boiling for 5 min, the relative conductivity of the treated bacteria solution was measured again (denoted as L0). The relative conductivity of the bacterial membrane permeability is calculated as: Relative conductivity (%) =100× (L2 -L1)/L0. Paired t-test was used to analyze the difference between BL21 (pET32a-mMn-SOD) and BL21 (pET32a) in each concentration group. The symbols * indicate statistical significance, *P* < 0.05. Values are expressed as means ±*S.E*. (*n*=3).
