# Supplementary material for: Characterization of a Mn-SOD from the desert beetle Microdera punctipennis and its increased resistance to cold stress in E. coli cells
Source: PeerJ. 2020 Feb 14;8:e8507. doi: 10.7717/peerj.8507 (PMC7025704; doi:10.7717/peerj.8507)
Supplement: Supplemental Information 7 — The MDA content was determined by using the MDA Assay Kit (Solarbio, Beijing, China) according to the manufacturer’s protocol. Paired t-test was used to analyze the difference between BL21 (pET32a-mMn-SOD) and BL21 (pET32a) in each concentration group. The symbols * indicate statistical significance, P < 0.05. Values are expressed as means ± S.E. (n = 3). [file peerj-08-8507-s007.docx]

|  | BL21(pET32a) | | | BL21(pET32a-Mn-SOD) | | |
| --- | --- | --- | --- | --- | --- | --- |
| 0h | 0.041 | 0.03511 | 0.02557 | 0.04044 | 0.03343 | 0.02978 |
| 2h | 0.03316 | 0.04017 | 0.02874 | 0.033 | 0.04033 | 0.03833 |
| 4h | 0.04633 | 0.05278 | 0.04279 | 0.03488 | 0.03888 | 0.03866 |
| 6h | 0.0612 | 0.06232 | 0.04633 | 0.0382 | 0.04454 | 0.04353 |

**Supplementary data. S7. MDA content data.** The MDA content was determined by using the MDA Assay Kit (Solarbio, Beijing, China) according to the manufacturer's protocol. Paired t-test was used to analyze the difference between BL21 (pET32a-mMn-SOD) and BL21 (pET32a) in each concentration group. The symbols * indicate statistical significance, *P* < 0.05. Values are expressed as means ±*S.E*. (*n*=3).
