# Supplementary material for: Characterization of a Mn-SOD from the desert beetle Microdera punctipennis and its increased resistance to cold stress in E. coli cells
Source: PeerJ. 2020 Feb 14;8:e8507. doi: 10.7717/peerj.8507 (PMC7025704; doi:10.7717/peerj.8507)
Supplement: Supplemental Information 8 — (A) mRNA, complete cds. (B) predicted amino acid sequence. [file peerj-08-8507-s008.docx]

A.

ATGTTTAAATTACCAAAATTACCTTATTCCTTTAATGCTTTAGCTCCACGTATATCTGCAGAAACATTACAGTTTCATTACAGTAAACATCATGCTGCATATGTAAATAAATTGAATGAAATTTTAAGAGACAAAAAGAGTAAGGATCAACCAAAATCTCTTTTGGAAATTATAAGAAAAGAAAATAATGGGACATTATTCAATCAAGCAGCTCAATCGTGGAATCATGAATTTTACTGGAATTGTCTAACCCCAAATGGAGGAGGTAAACCATACGGACTAGTACAAACATTAATTGAGCGTGACTATCAAGATTTTGATACGTTTAAAAATCAATTTACAAGTGCAGCATCCACGCATTTTGGTTCAGGATGGGTATGGTTATGTTTTAATAAAGAAAGTGGAAAACTTGAGATCCAA CAAACACATGATGCCCAACATCCAATTAAATTAAATTCTAATTTGGTGCCAATATTGACTTGTGACATTTGGGAACATGCTTATTATATAGACTATAGAAATGCTCGACCAAAATATATACAAGCTTGGTGGGATACATTGAACTGGAATTTTGCTAATTTATGTTTGGAAAAAAAATCCGTCGACTAAGAGTTAATATAAATTTTTTTCTTTTTTGCTTTTTTCTATCTGCATGCAACGCTGAAATTAGTCAACAAATAAGCTATTTTTTTCATTATATAATTTTTATAGATAACCAACTTTTTTAACAATTCTAGAAACAAATGGTTTTTGAATTAGCAAAATTACCTTTTAGCGAAGATGCGCTTAAACCGCATATATCCCCTCAAACATTACAATTTCATCATGGCAAACATCATGCGTCATATGTTAATAATTTGAACAATCTAACTAAAGGGACACCAATGGAACGCTTAAGCTTACAAGATGTTGTCATCCAAGCTGAAGGTGCAACATTTAACCAAGCGGCACAAGCATGGAATCATGATTTTTATTGGCAATCTTTAACCCCGTCTCAATCTGGTGGACCAATAGGAGAGCTTAAACAAATGATAGAAGCAGAATTTGGAAATTTTGATGAATTTAAAAGTAAGTTTTCTAGTGCAGCTTCAGGACATTTTGGCTCCGGATGGGCATGGCTTGTCTATGATACTAAACAAAATAAAGTTAAAATACATCAAACACATGACGCAGGAAATCCATTAAAGGATGGAGCTGGAATTCCACTATTGACATGTGATGTATGGGAACATGCATATTATCTCGATTATCAGAATAATAGAGCTCAATATATTGACGCTTGGTGGAAAATAGCCAATTGGAAATTTGCAGAAGAAAATTTAAGAAAGTGCTTGAAAAAATAAACTATTAAAAAAAAAA

B.

MFKLPKLPYSFNALAPRISAETLQFHYSKHHAAYVNKLNEILRDKKSKDQPKSLLEIIRKENNGTLFNQAAQSWNHEFYWNCLTPNGGGKPYGLVQTLIERDYQDFDTFKNQFTSAASTHFGSGWVWLCFNKESGKLEIQQTHDAQHPIKLNSNLVPILTCDIWEHAYYIDYRNARPKYIQAWWDTLNWNFANLCLEKKSVD

Supplementary data. S8. *Microdera punctipennis* manganese superoxide dismutase sequence. (A) mRNA, complete cds. (B) predicted amino acid sequence.
