# Supplementary material for: Characterization of a Mn-SOD from the desert beetle Microdera punctipennis and its increased resistance to cold stress in E. coli cells
Source: PeerJ. 2020 Feb 14;8:e8507. doi: 10.7717/peerj.8507 (PMC7025704; doi:10.7717/peerj.8507)
Supplement: Supplemental Information 9 — The survival of E. coli BL21(pET32a), E. coli BL21(pET32a-MpmMn-SOD) after exposed to −4 °C for different time lengths (2 h, 4 h, 6 h and 8h) were measured by OD595. The control (0h) was kept at 25 °C without cold treatment. Values are expressed as means ± S.D. (n = 3). [file peerj-08-8507-s009.docx]

|  | BL21(PET32a) | | | BL21(PET32a-mMn SOD) | | |
| --- | --- | --- | --- | --- | --- | --- |
| 0h | 0.563 | 0.586 | 0.587 | 0.569 | 0.579 | 0.597 |
| 2h | 0.531 | 0.548 | 0.543 | 0.613 | 0.618 | 0.604 |
| 4h | 0.524 | 0.516 | 0.512 | 0.639 | 0.614 | 0.621 |
| 6h | 0.511 | 0.483 | 0.508 | 0.573 | 0.594 | 0.587 |
| 8h | 0.497 | 0.481 | 0.476 | 0.554 | 0.561 | 0.547 |

Supplementary data. S9. OD value (OD595) of *E. coli* BL21(pET32a) and *E. coli* BL21(pET32a-MpmMn-SOD) after cold exposure for different time. The survival of *E. coli* BL21(pET32a), *E. coli* BL21(pET32a-MpmMn-SOD) after exposed to -4 ℃ for different time lengths (2 h, 4 h, 6 h and 8h) were measured by OD595. The control (0h) was kept at 25℃ without cold treatment. Values are expressed as means ± *S.D*. (*n*=3).
